# miRNA-429 suppresses osteogenic differentiation of human adipose-derived mesenchymal stem cells under oxidative stress via targeting SCD-1

**DOI:** 10.3892/etm.2022.11311

**Published:** 2022-04-11

**Authors:** Changgong Lan, Lizhen Long, Kegong Xie, Jia Liu, Landao Zhou, Shengcai Pan, Junqing Liang, Zhenyang Tu, Ziran Gao, Yujin Tang

Exp Ther Med 19:696–702, 2020; DOI: 10.3892/etm.2019.8246

Following the publication of this article, a concerned reader drew to our attention that a pair of the data panels featured in Fig. 4D featured some striking and unexpected similarities with data panels in Fig. 2D in an article published at a similar time [Luo B, Yang J-F, Wang Y-H, Qu G-B, Hao P-D, Zeng Z-J, Yuan J and Yuan Y: MicroRNA-579-3p promotes the progression of osteoporosis by inhibiting osteogenic differentiation of mesenchymal stem cells through regulating Sirt1. Eur Rev Med Pharmacol Sci 23: 6791-6799, 2019], and Fig. 2E in another article that was published in 2019 [Fy Y-C, Zhao S-R, Zhu B-H, Guo S-S and Wang X-X: MiRNA-27a-3p promotes osteogenic differentiation of human mesenchymal stem cells through targeting ATF3. Eur Rev Med Pharmacol Sci 23: 73-80, 2019].

After having conducted an internal investigation, the Editor of *Experimental and Therapeutic Medicine* agrees that the data in question had originated from the same original source(s). Therefore, owing to the fact that the contentious data in the above article had already been published elsewhere, or were already under consideration for publication, prior to its submission to *Experimental and Therapeutic Medicine*, the Editor has decided that this paper should be retracted from the Journal. The authors independently contacted the Editorial Office to request that the article be retracted on account of an authorship dispute. The Editor sincerely apologizes to the readership for the inconvenience caused, and we also thank the reader for bringing this matter to our attention.

